# Practical Points

**Published:** 1896-12-05

**Authors:** 


### PRACTICAL POINTS.

"*Treating a Pine Wood Floor.*"—Mr. Walter Maguire sends the following suggestion in reply to a recent query on the subject: In reply to the above inquiry, first cleanse the floor with warm water (to which a little liq. ammonia has been added), and sponge, removing carefully all grit and grease; when perfectly dry, rub in by hand with a wad of tow raw linseed oil, containing 1 in 5 of turps, using the mixture liberally, and letting it run well into the seams. This will be absorbed in about twelve hours if the room is warm, when a coat of hard-drying oak varnish should be given, care being taken to first rub off any unabsorbed oil, and to keep a good fire up until dry. If the varnish is bought from a good maker (and Mander Brothers, of Wolverhampton and London, supply an excellent varnish at 12s. 6d. per gallon) it should be hard in six hours, and should then be sponged over with cold water, and a second coat applied. A week should be allowed free from traffic, and, if sponged daily with cold water, it would be as hard as marble, and wear for years. Linseed oil with a little ol. eucalyptus added, used sparingly and briskly, is a better polisher for varnished floors than beeswax; it is applied in a third of the time, is cheaper, and is never "tacky" to the tread. I shall be pleased to give your correspondent any further information. I may add that a gallon of good varnish used in a warm temperature will cover a floor 80 ft. by 30 ft. twice. To keep a floor in good condition the varnish should never be permitted to wear off down to the wood, otherwise it will darken and look patchy, and can never afterwards be made to look well. A brush of varnish can be thinly applied here and there in a few minutes.

